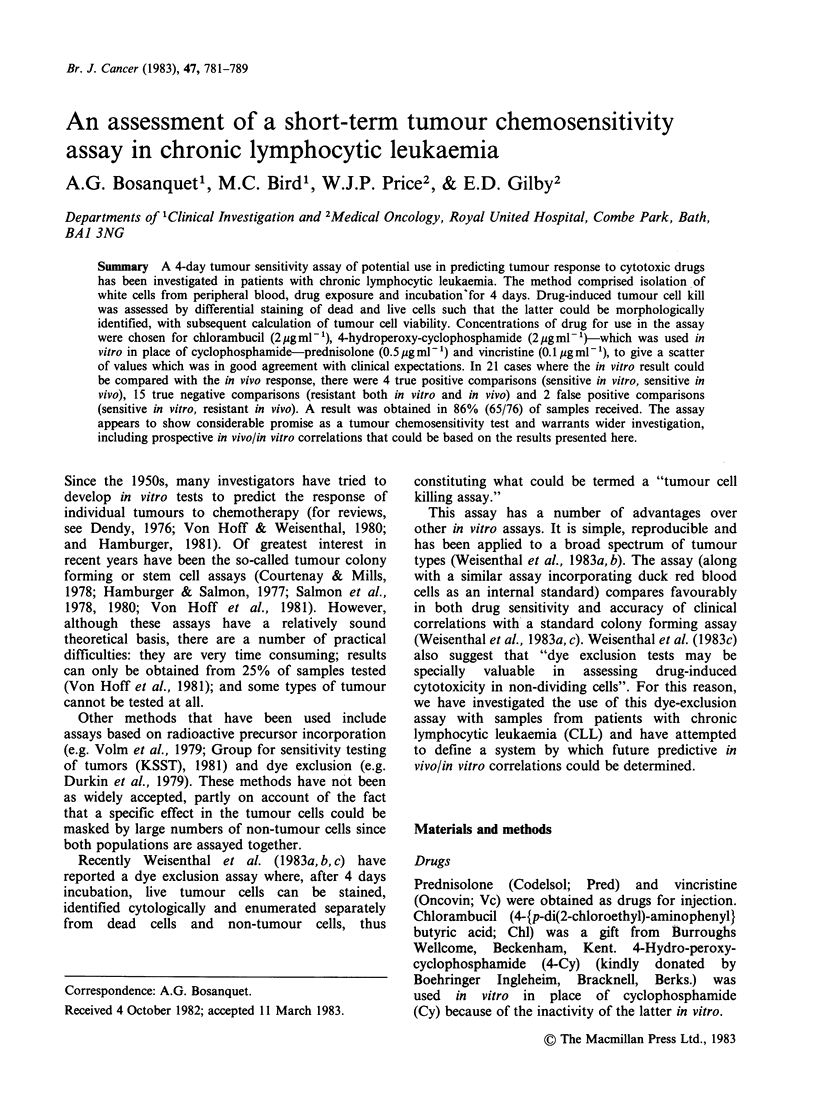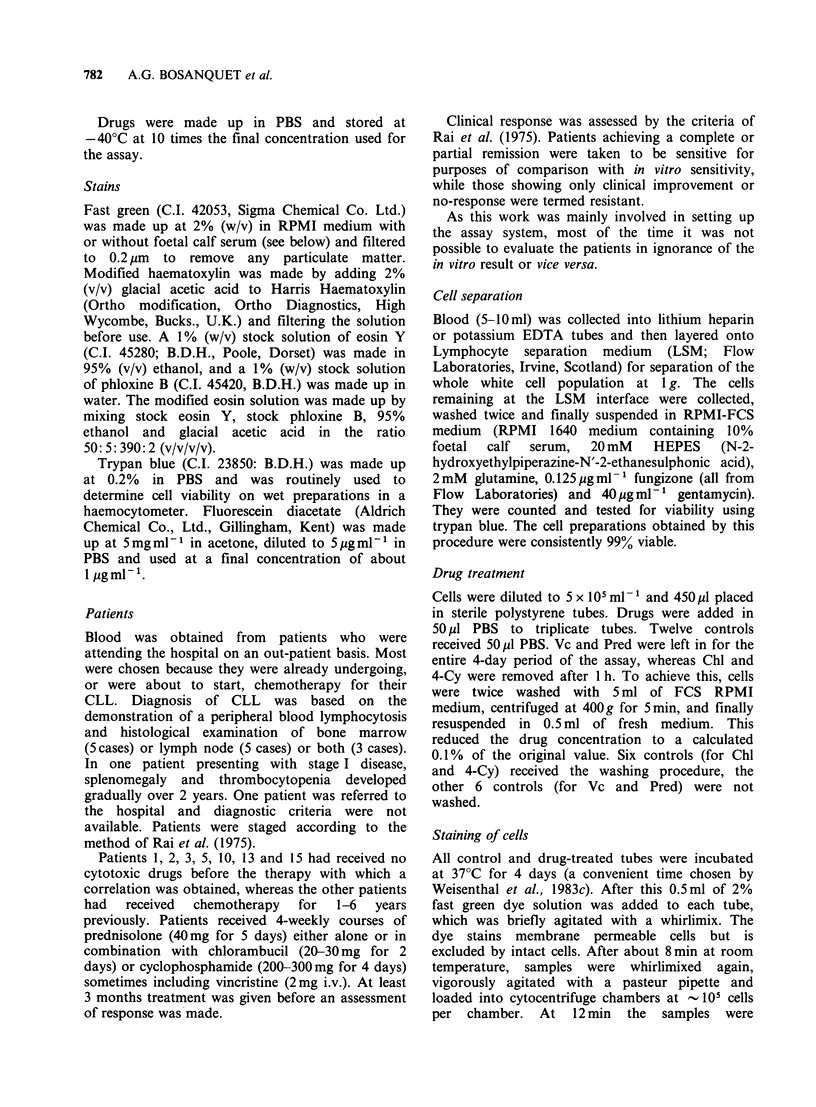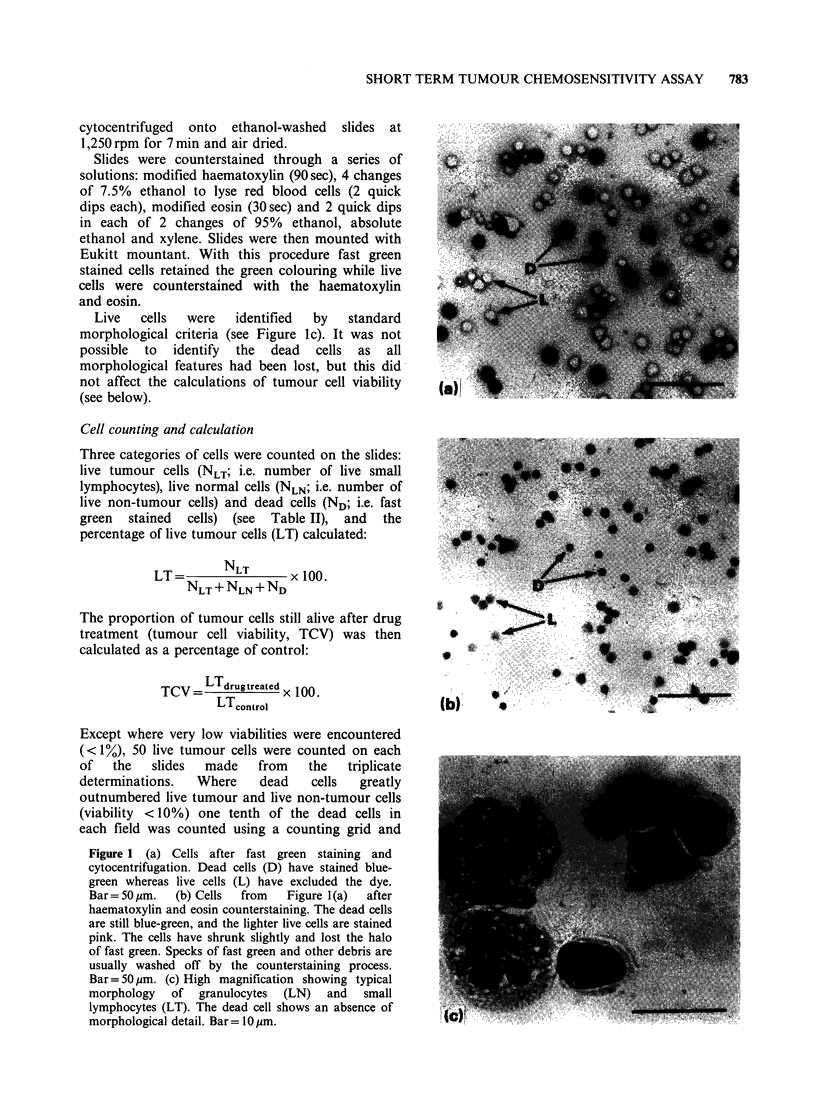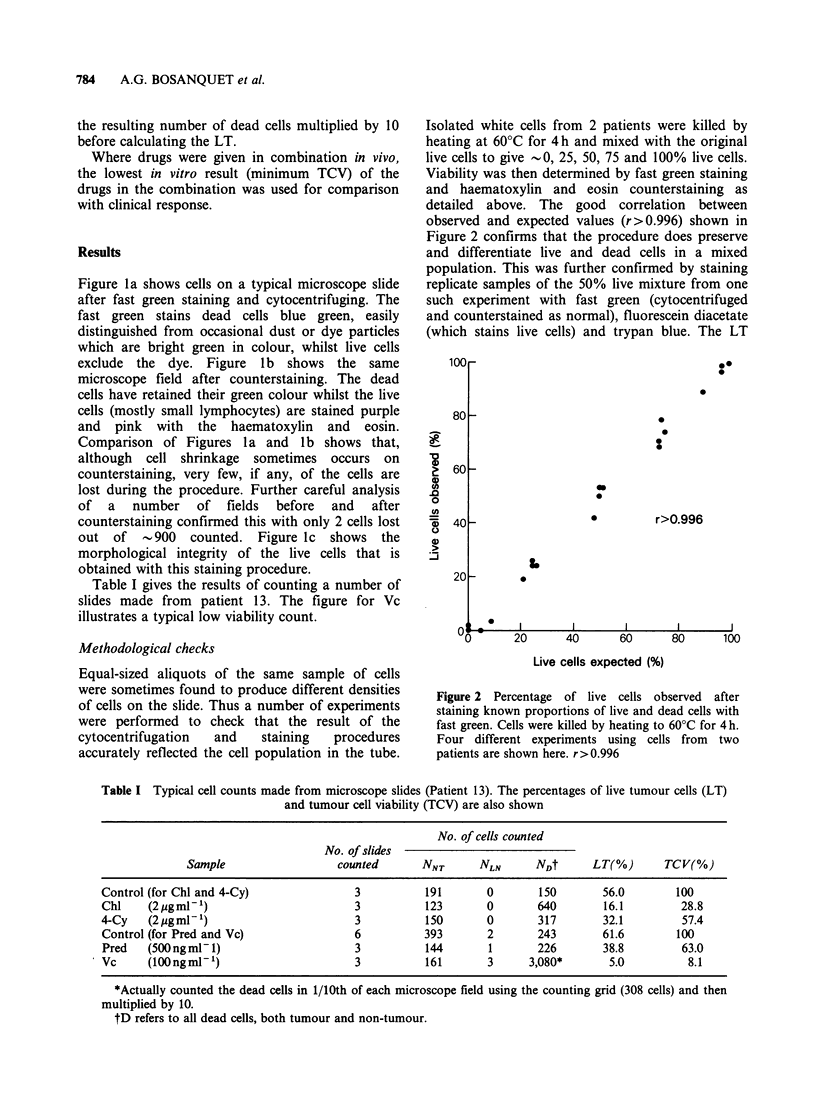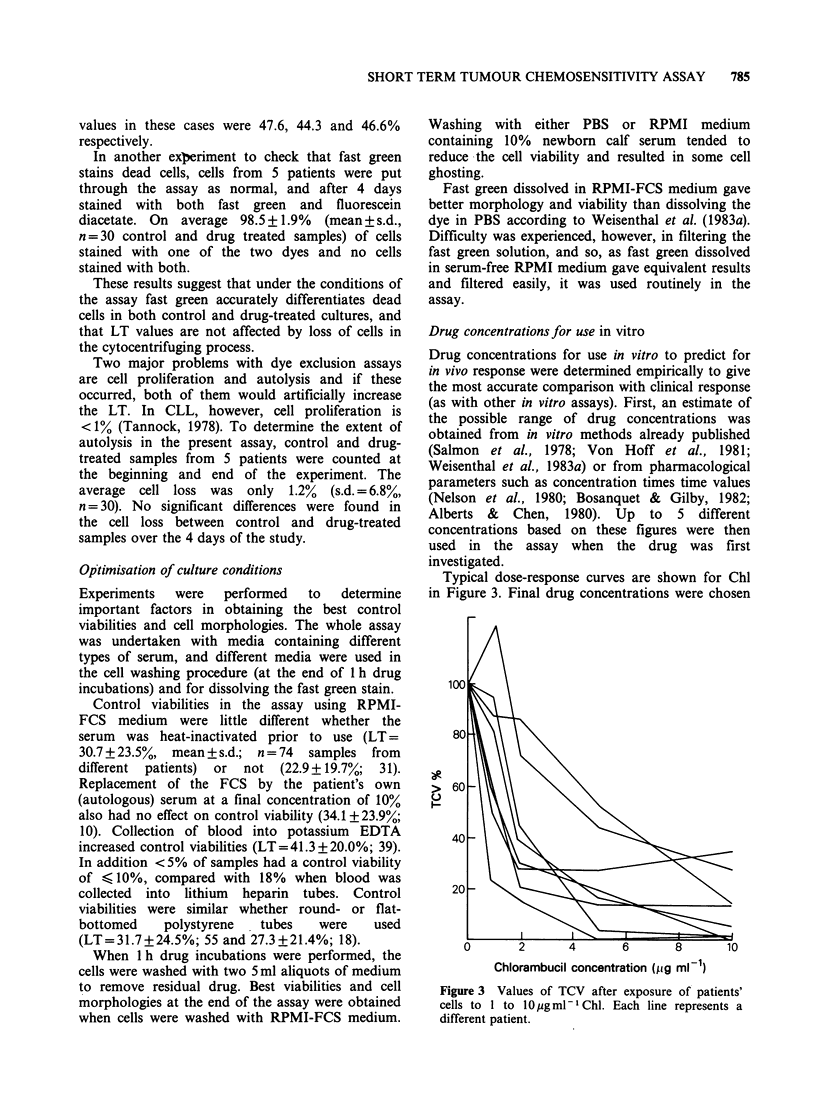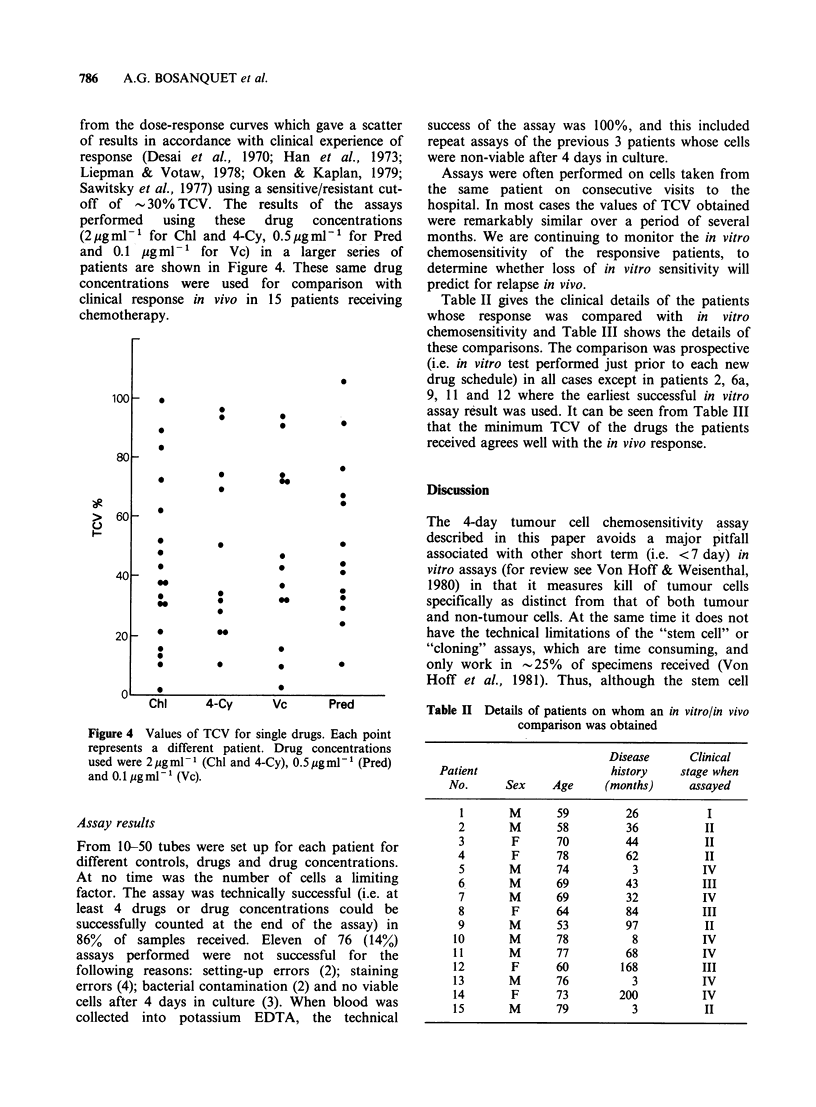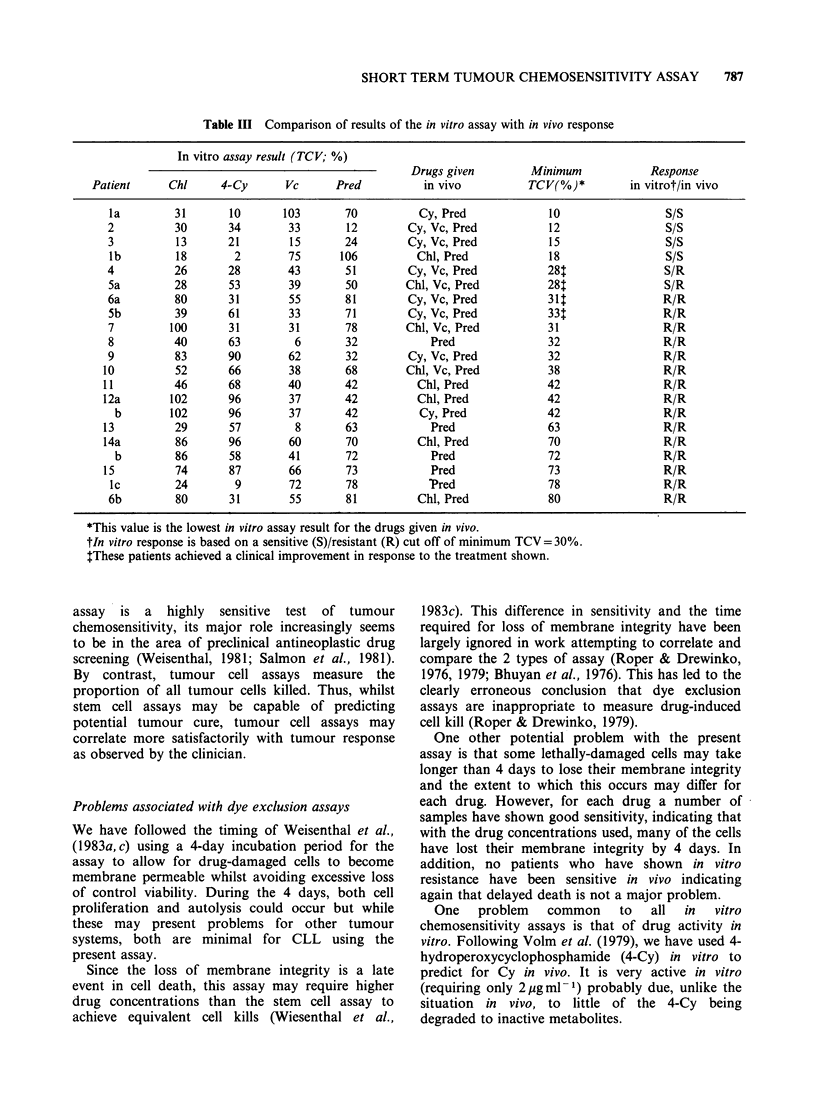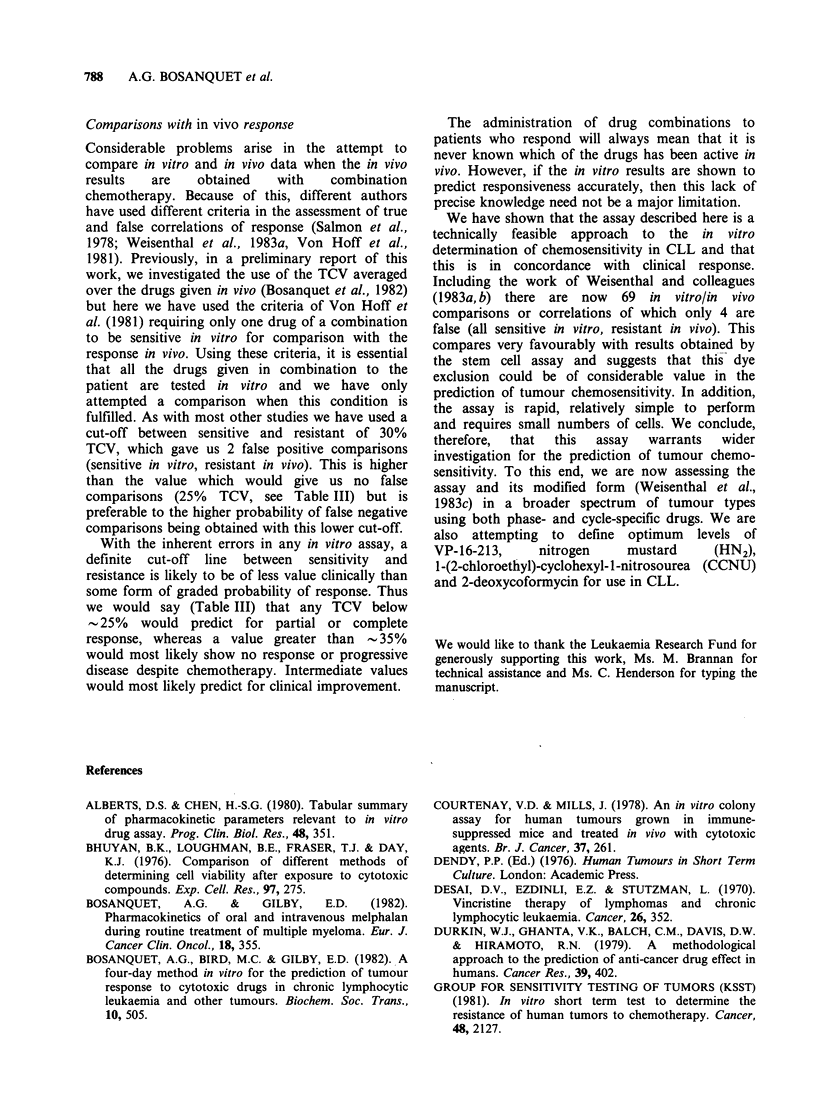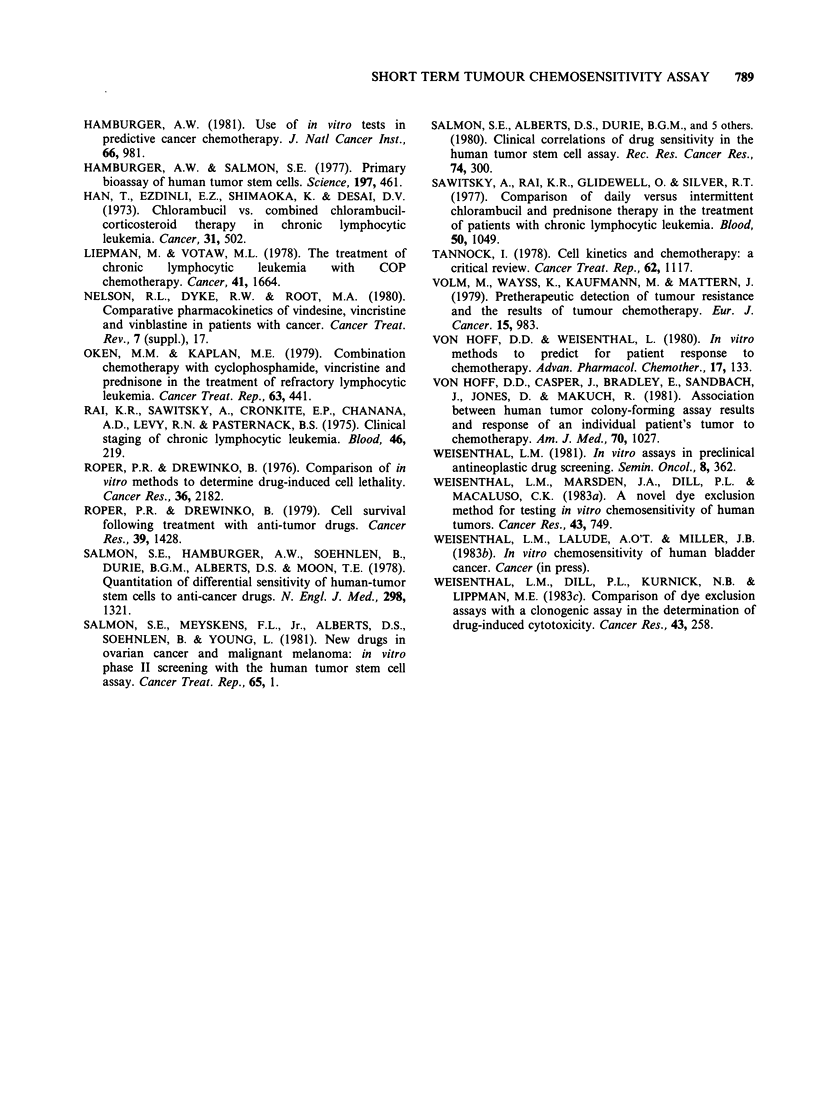# An assessment of a short-term tumour chemosensitivity assay in chronic lymphocytic leukaemia.

**DOI:** 10.1038/bjc.1983.131

**Published:** 1983-06

**Authors:** A. G. Bosanquet, M. C. Bird, W. J. Price, E. D. Gilby

## Abstract

**Images:**


*Br. J. Cancer* (1983), **47**, 781–789

### An assessment of a short-term tumour chemosensitivity assay in chronic lymphocytic leukaemia

A.G. Bosanquet[1], M.C. Bird[1], W.J.P. Price[2], & E.D. Gilby[2]

*Departments of [1]Clinical Investigation and [2]Medical Oncology, Royal United Hospital, Combe Park, Bath, BA1 3NG*

**Summary** A 4-day tumour sensitivity assay of potential use in predicting tumour response to cytotoxic drugs has been investigated in patients with chronic lymphocytic leukaemia. The method comprised isolation of white cells from peripheral blood, drug exposure and incubation for 4 days. Drug-induced tumour cell kill was assessed by differential staining of dead and live cells such that the latter could be morphologically identified, with subsequent calculation of tumour cell viability. Concentrations of drug for use in the assay were chosen for chlorambucil (2 $\mu g\,ml^{-1}$), 4-hydroperoxy-cyclophosphamide (2 $\mu g\,ml^{-1}$)—which was used *in vitro* in place of cyclophosphamide—prednisolone (0.5 $\mu g\,ml^{-1}$) and vincristine (0.1 $\mu g\,ml^{-1}$), to give a scatter of values which was in good agreement with clinical expectations. In 21 cases where the *in vitro* result could be compared with the *in vivo* response, there were 4 true positive comparisons (sensitive *in vitro*, sensitive *in vivo*), 15 true negative comparisons (resistant both *in vitro* and *in vivo*) and 2 false positive comparisons (sensitive *in vitro*, resistant *in vivo*). A result was obtained in 86% (65/76) of samples received. The assay appears to show considerable promise as a tumour chemosensitivity test and warrants wider investigation, including prospective *in vivo*/*in vitro* correlations that could be based on the results presented here.

Since the 1950s, many investigators have tried to develop *in vitro* tests to predict the response of individual tumours to chemotherapy (for reviews, see Dendy, 1976; Von Hoff & Weisenthal, 1980; and Hamburger, 1981). Of greatest interest in recent years have been the so-called tumour colony forming or stem cell assays (Courtenay & Mills, 1978; Hamburger & Salmon, 1977; Salmon *et al.*, 1978, 1980; Von Hoff *et al.*, 1981). However, although these assays have a relatively sound theoretical basis, there are a number of practical difficulties: they are very time consuming; results can only be obtained from 25% of samples tested (Von Hoff *et al.*, 1981); and some types of tumour cannot be tested at all.

Other methods that have been used include assays based on radioactive precursor incorporation (e.g. Volm *et al.*, 1979; Group for sensitivity testing of tumors (KSST), 1981) and dye exclusion (e.g. Durkin *et al.*, 1979). These methods have not been as widely accepted, partly on account of the fact that a specific effect in the tumour cells could be masked by large numbers of non-tumour cells since both populations are assayed together.

Recently Weisenthal *et al.* (1983*a, b, c*) have reported a dye exclusion assay where, after 4 days incubation, live tumour cells can be stained, identified cytologically and enumerated separately from dead cells and non-tumour cells, thus constituting what could be termed a "tumour cell killing assay."

This assay has a number of advantages over other *in vitro* assays. It is simple, reproducible and has been applied to a broad spectrum of tumour types (Weisenthal *et al.*, 1983*a, b*). The assay (along with a similar assay incorporating duck red blood cells as an internal standard) compares favourably in both drug sensitivity and accuracy of clinical correlations with a standard colony forming assay (Weisenthal *et al.*, 1983*a, c*). Weisenthal *et al.* (1983*c*) also suggest that "dye exclusion tests may be specially valuable in assessing drug-induced cytotoxicity in non-dividing cells". For this reason, we have investigated the use of this dye-exclusion assay with samples from patients with chronic lymphocytic leukaemia (CLL) and have attempted to define a system by which future predictive *in vivo*/*in vitro* correlations could be determined.

### Materials and methods

### *Drugs*

Prednisolone (Codelsol; Pred) and vincristine (Oncovin; Vc) were obtained as drugs for injection. Chlorambucil (4-{*p*-di(2-chloroethyl)-aminophenyl} butyric acid; Chl) was a gift from Burroughs Wellcome, Beckenham, Kent. 4-Hydro-peroxy-cyclophosphamide (4-Cy) (kindly donated by Boehringer Ingleheim, Bracknell, Berks.) was used *in vitro* in place of cyclophosphamide (Cy) because of the inactivity of the latter *in vitro*.

Correspondence: A.G. Bosanquet.

Received 4 October 1982; accepted 11 March 1983.

© The Macmillan Press Ltd., 1983

Drugs were made up in PBS and stored at −40°C at 10 times the final concentration used for the assay.

*Stains*

Fast green (C.I. 42053, Sigma Chemical Co. Ltd.) was made up at 2% (w/v) in RPMI medium with or without foetal calf serum (see below) and filtered to 0.2 μm to remove any particulate matter. Modified haematoxylin was made by adding 2% (v/v) glacial acetic acid to Harris Haematoxylin (Ortho modification, Ortho Diagnostics, High Wycombe, Bucks., U.K.) and filtering the solution before use. A 1% (w/v) stock solution of eosin Y (C.I. 45280; B.D.H., Poole, Dorset) was made in 95% (v/v) ethanol, and a 1% (w/v) stock solution of phloxine B (C.I. 45420, B.D.H.) was made up in water. The modified eosin solution was made up by mixing stock eosin Y, stock phloxine B, 95% ethanol and glacial acetic acid in the ratio 50:5:390:2 (v/v/v/v).

Trypan blue (C.I. 23850: B.D.H.) was made up at 0.2% in PBS and was routinely used to determine cell viability on wet preparations in a haemocytometer. Fluorescein diacetate (Aldrich Chemical Co., Ltd., Gillingham, Kent) was made up at 5 mg ml$^{-1}$ in acetone, diluted to 5 μg ml$^{-1}$ in PBS and used at a final concentration of about 1 μg ml$^{-1}$.

*Patients*

Blood was obtained from patients who were attending the hospital on an out-patient basis. Most were chosen because they were already undergoing, or were about to start, chemotherapy for their CLL. Diagnosis of CLL was based on the demonstration of a peripheral blood lymphocytosis and histological examination of bone marrow (5 cases) or lymph node (5 cases) or both (3 cases). In one patient presenting with stage I disease, splenomegaly and thrombocytopenia developed gradually over 2 years. One patient was referred to the hospital and diagnostic criteria were not available. Patients were staged according to the method of Rai *et al.* (1975).

Patients 1, 2, 3, 5, 10, 13 and 15 had received no cytotoxic drugs before the therapy with which a correlation was obtained, whereas the other patients had received chemotherapy for 1–6 years previously. Patients received 4-weekly courses of prednisolone (40 mg for 5 days) either alone or in combination with chlorambucil (20–30 mg for 2 days) or cyclophosphamide (200–300 mg for 4 days) sometimes including vincristine (2 mg i.v.). At least 3 months treatment was given before an assessment of response was made.

Clinical response was assessed by the criteria of Rai *et al.* (1975). Patients achieving a complete or partial remission were taken to be sensitive for purposes of comparison with *in vitro* sensitivity, while those showing only clinical improvement or no-response were termed resistant.

As this work was mainly involved in setting up the assay system, most of the time it was not possible to evaluate the patients in ignorance of the *in vitro* result or *vice versa.*

*Cell separation*

Blood (5–10 ml) was collected into lithium heparin or potassium EDTA tubes and then layered onto Lymphocyte separation medium (LSM; Flow Laboratories, Irvine, Scotland) for separation of the whole white cell population at 1 *g*. The cells remaining at the LSM interface were collected, washed twice and finally suspended in RPMI-FCS medium (RPMI 1640 medium containing 10% foetal calf serum, 20 mM HEPES (N-2-hydroxyethylpiperazine-N′-2-ethanesulphonic acid), 2 mM glutamine, 0.125 μg ml$^{-1}$ fungizone (all from Flow Laboratories) and 40 μg ml$^{-1}$ gentamycin). They were counted and tested for viability using trypan blue. The cell preparations obtained by this procedure were consistently 99% viable.

*Drug treatment*

Cells were diluted to $5 \times 10^5$ ml$^{-1}$ and 450 μl placed in sterile polystyrene tubes. Drugs were added in 50 μl PBS to triplicate tubes. Twelve controls received 50 μl PBS. Vc and Pred were left in for the entire 4-day period of the assay, whereas Chl and 4-Cy were removed after 1 h. To achieve this, cells were twice washed with 5 ml of FCS RPMI medium, centrifuged at 400 *g* for 5 min, and finally resuspended in 0.5 ml of fresh medium. This reduced the drug concentration to a calculated 0.1% of the original value. Six controls (for Chl and 4-Cy) received the washing procedure, the other 6 controls (for Vc and Pred) were not washed.

*Staining of cells*

All control and drug-treated tubes were incubated at 37°C for 4 days (a convenient time chosen by Weisenthal *et al.*, 1983*c*). After this 0.5 ml of 2% fast green dye solution was added to each tube, which was briefly agitated with a whirlimix. The dye stains membrane permeable cells but is excluded by intact cells. After about 8 min at room temperature, samples were whirlimixed again, vigorously agitated with a pasteur pipette and loaded into cytocentrifuge chambers at $\sim 10^5$ cells per chamber. At 12 min the samples were cytocentrifuged onto ethanol-washed slides at 1,250 rpm for 7 min and air dried.

Slides were counterstained through a series of solutions: modified haematoxylin (90 sec), 4 changes of 7.5% ethanol to lyse red blood cells (2 quick dips each), modified eosin (30 sec) and 2 quick dips in each of 2 changes of 95% ethanol, absolute ethanol and xylene. Slides were then mounted with Eukitt mountant. With this procedure fast green stained cells retained the green colouring while live cells were counterstained with the haematoxylin and eosin.

Live cells were identified by standard morphological criteria (see Figure 1c). It was not possible to identify the dead cells as all morphological features had been lost, but this did not affect the calculations of tumour cell viability (see below).

*Cell counting and calculation*

Three categories of cells were counted on the slides: live tumour cells ($N_{LT}$; i.e. number of live small lymphocytes), live normal cells ($N_{LN}$; i.e. number of live non-tumour cells) and dead cells ($N_D$; i.e. fast green stained cells) (see Table II), and the percentage of live tumour cells (LT) calculated:

$$LT = \frac{N_{LT}}{N_{LT} + N_{LN} + N_D} \times 100.$$

The proportion of tumour cells still alive after drug treatment (tumour cell viability, TCV) was then calculated as a percentage of control:

$$TCV = \frac{LT_{drug\,treated}}{LT_{control}} \times 100.$$

Except where very low viabilities were encountered (<1%), 50 live tumour cells were counted on each of the slides made from the triplicate determinations. Where dead cells greatly outnumbered live tumour and live non-tumour cells (viability <10%) one tenth of the dead cells in each field was counted using a counting grid and

**Figure 1** (a) Cells after fast green staining and cytocentrifugation. Dead cells (D) have stained blue-green whereas live cells (L) have excluded the dye. Bar = 50 μm. (b) Cells from Figure 1(a) after haematoxylin and eosin counterstaining. The dead cells are still blue-green, and the lighter live cells are stained pink. The cells have shrunk slightly and lost the halo of fast green. Specks of fast green and other debris are usually washed off by the counterstaining process. Bar = 50 μm. (c) High magnification showing typical morphology of granulocytes (LN) and small lymphocytes (LT). The dead cell shows an absence of morphological detail. Bar = 10 μm.

the resulting number of dead cells multiplied by 10 before calculating the LT.

Where drugs were given in combination *in vivo*, the lowest *in vitro* result (minimum TCV) of the drugs in the combination was used for comparison with clinical response.

### Results

Figure 1a shows cells on a typical microscope slide after fast green staining and cytocentrifuging. The fast green stains dead cells blue green, easily distinguished from occasional dust or dye particles which are bright green in colour, whilst live cells exclude the dye. Figure 1b shows the same microscope field after counterstaining. The dead cells have retained their green colour whilst the live cells (mostly small lymphocytes) are stained purple and pink with the haematoxylin and eosin. Comparison of Figures 1a and 1b shows that, although cell shrinkage sometimes occurs on counterstaining, very few, if any, of the cells are lost during the procedure. Further careful analysis of a number of fields before and after counterstaining confirmed this with only 2 cells lost out of ~900 counted. Figure 1c shows the morphological integrity of the live cells that is obtained with this staining procedure.

Table I gives the results of counting a number of slides made from patient 13. The figure for Vc illustrates a typical low viability count.

### *Methodological checks*

Equal-sized aliquots of the same sample of cells were sometimes found to produce different densities of cells on the slide. Thus a number of experiments were performed to check that the result of the cytocentrifugation and staining procedures accurately reflected the cell population in the tube. Isolated white cells from 2 patients were killed by heating at 60°C for 4 h and mixed with the original live cells to give ~0, 25, 50, 75 and 100% live cells. Viability was then determined by fast green staining and haematoxylin and eosin counterstaining as detailed above. The good correlation between observed and expected values ($r > 0.996$) shown in Figure 2 confirms that the procedure does preserve and differentiate live and dead cells in a mixed population. This was further confirmed by staining replicate samples of the 50% live mixture from one such experiment with fast green (cytocentrifuged and counterstained as normal), fluorescein diacetate (which stains live cells) and trypan blue. The LT

**Figure 2** Percentage of live cells observed after staining known proportions of live and dead cells with fast green. Cells were killed by heating to 60°C for 4 h. Four different experiments using cells from two patients are shown here. $r > 0.996$

**Table I** Typical cell counts made from microscope slides (Patient 13). The percentages of live tumour cells (LT) and tumour cell viability (TCV) are also shown

| *Sample* | *No. of slides counted* | *No. of cells counted* $N_{NT}$ | $N_{LN}$ | $N_D$† | *LT(%)* | *TCV(%)* |
|---|---|---|---|---|---|---|
| Control (for Chl and 4-Cy) | 3 | 191 | 0 | 150 | 56.0 | 100 |
| Chl (2 μg ml$^{-1}$) | 3 | 123 | 0 | 640 | 16.1 | 28.8 |
| 4-Cy (2 μg ml$^{-1}$) | 3 | 150 | 0 | 317 | 32.1 | 57.4 |
| Control (for Pred and Vc) | 6 | 393 | 2 | 243 | 61.6 | 100 |
| Pred (500 ng ml$^{-}$1) | 3 | 144 | 1 | 226 | 38.8 | 63.0 |
| Vc (100 ng ml$^{-1}$) | 3 | 161 | 3 | 3,080* | 5.0 | 8.1 |

*Actually counted the dead cells in 1/10th of each microscope field using the counting grid (308 cells) and then multiplied by 10.

†D refers to all dead cells, both tumour and non-tumour.

values in these cases were 47.6, 44.3 and 46.6% respectively.

In another experiment to check that fast green stains dead cells, cells from 5 patients were put through the assay as normal, and after 4 days stained with both fast green and fluorescein diacetate. On average 98.5±1.9% (mean±s.d., $n=30$ control and drug treated samples) of cells stained with one of the two dyes and no cells stained with both.

These results suggest that under the conditions of the assay fast green accurately differentiates dead cells in both control and drug-treated cultures, and that LT values are not affected by loss of cells in the cytocentrifuging process.

Two major problems with dye exclusion assays are cell proliferation and autolysis and if these occurred, both of them would artificially increase the LT. In CLL, however, cell proliferation is <1% (Tannock, 1978). To determine the extent of autolysis in the present assay, control and drug-treated samples from 5 patients were counted at the beginning and end of the experiment. The average cell loss was only 1.2% (s.d.=6.8%, $n=30$). No significant differences were found in the cell loss between control and drug-treated samples over the 4 days of the study.

*Optimisation of culture conditions*

Experiments were performed to determine important factors in obtaining the best control viabilities and cell morphologies. The whole assay was undertaken with media containing different types of serum, and different media were used in the cell washing procedure (at the end of 1 h drug incubations) and for dissolving the fast green stain.

Control viabilities in the assay using RPMI-FCS medium were little different whether the serum was heat-inactivated prior to use (LT=30.7±23.5%, mean±s.d.; $n=74$ samples from different patients) or not (22.9±19.7%; 31). Replacement of the FCS by the patient's own (autologous) serum at a final concentration of 10% also had no effect on control viability (34.1±23.9%; 10). Collection of blood into potassium EDTA increased control viabilities (LT=41.3±20.0%; 39). In addition <5% of samples had a control viability of ⩽10%, compared with 18% when blood was collected into lithium heparin tubes. Control viabilities were similar whether round- or flat-bottomed polystyrene tubes were used (LT=31.7±24.5%; 55 and 27.3±21.4%; 18).

When 1 h drug incubations were performed, the cells were washed with two 5 ml aliquots of medium to remove residual drug. Best viabilities and cell morphologies at the end of the assay were obtained when cells were washed with RPMI-FCS medium. Washing with either PBS or RPMI medium containing 10% newborn calf serum tended to reduce the cell viability and resulted in some cell ghosting.

Fast green dissolved in RPMI-FCS medium gave better morphology and viability than dissolving the dye in PBS according to Weisenthal *et al.* (1983*a*). Difficulty was experienced, however, in filtering the fast green solution, and so, as fast green dissolved in serum-free RPMI medium gave equivalent results and filtered easily, it was used routinely in the assay.

*Drug concentrations for use* in vitro

Drug concentrations for use *in vitro* to predict for *in vivo* response were determined empirically to give the most accurate comparison with clinical response (as with other *in vitro* assays). First, an estimate of the possible range of drug concentrations was obtained from *in vitro* methods already published (Salmon *et al.*, 1978; Von Hoff *et al.*, 1981; Weisenthal *et al.*, 1983*a*) or from pharmacological parameters such as concentration times time values (Nelson *et al.*, 1980; Bosanquet & Gilby, 1982; Alberts & Chen, 1980). Up to 5 different concentrations based on these figures were then used in the assay when the drug was first investigated.

Typical dose-response curves are shown for Chl in Figure 3. Final drug concentrations were chosen

**Figure 3** Values of TCV after exposure of patients' cells to 1 to 10 μg ml$^{-1}$ Chl. Each line represents a different patient.

from the dose-response curves which gave a scatter of results in accordance with clinical experience of response (Desai *et al.*, 1970; Han *et al.*, 1973; Liepman & Votaw, 1978; Oken & Kaplan, 1979; Sawitsky *et al.*, 1977) using a sensitive/resistant cut-off of ~30% TCV. The results of the assays performed using these drug concentrations ($2\,\mu g\,ml^{-1}$ for Chl and 4-Cy, $0.5\,\mu g\,ml^{-1}$ for Pred and $0.1\,\mu g\,ml^{-1}$ for Vc) in a larger series of patients are shown in Figure 4. These same drug concentrations were used for comparison with clinical response *in vivo* in 15 patients receiving chemotherapy.

**Figure 4** Values of TCV for single drugs. Each point represents a different patient. Drug concentrations used were $2\,\mu g\,ml^{-1}$ (Chl and 4-Cy), $0.5\,\mu g\,ml^{-1}$ (Pred) and $0.1\,\mu g\,ml^{-1}$ (Vc).

*Assay results*

From 10–50 tubes were set up for each patient for different controls, drugs and drug concentrations. At no time was the number of cells a limiting factor. The assay was technically successful (i.e. at least 4 drugs or drug concentrations could be successfully counted at the end of the assay) in 86% of samples received. Eleven of 76 (14%) assays performed were not successful for the following reasons: setting-up errors (2); staining errors (4); bacterial contamination (2) and no viable cells after 4 days in culture (3). When blood was collected into potassium EDTA, the technical success of the assay was 100%, and this included repeat assays of the previous 3 patients whose cells were non-viable after 4 days in culture.

Assays were often performed on cells taken from the same patient on consecutive visits to the hospital. In most cases the values of TCV obtained were remarkably similar over a period of several months. We are continuing to monitor the *in vitro* chemosensitivity of the responsive patients, to determine whether loss of *in vitro* sensitivity will predict for relapse *in vivo*.

Table II gives the clinical details of the patients whose response was compared with *in vitro* chemosensitivity and Table III shows the details of these comparisons. The comparison was prospective (i.e. *in vitro* test performed just prior to each new drug schedule) in all cases except in patients 2, 6a, 9, 11 and 12 where the earliest successful *in vitro* assay result was used. It can be seen from Table III that the minimum TCV of the drugs the patients received agrees well with the *in vivo* response.

### Discussion

The 4-day tumour cell chemosensitivity assay described in this paper avoids a major pitfall associated with other short term (i.e. <7 day) *in vitro* assays (for review see Von Hoff & Weisenthal, 1980) in that it measures kill of tumour cells specifically as distinct from that of both tumour and non-tumour cells. At the same time it does not have the technical limitations of the "stem cell" or "cloning" assays, which are time consuming, and only work in ~25% of specimens received (Von Hoff *et al.*, 1981). Thus, although the stem cell

**Table II** Details of patients on whom an *in vitro*/*in vivo* comparison was obtained

| *Patient No.* | *Sex* | *Age* | *Disease history (months)* | *Clinical stage when assayed* |
|---|---|---|---|---|
| 1 | M | 59 | 26 | I |
| 2 | M | 58 | 36 | II |
| 3 | F | 70 | 44 | II |
| 4 | F | 78 | 62 | II |
| 5 | M | 74 | 3 | IV |
| 6 | M | 69 | 43 | III |
| 7 | M | 69 | 32 | IV |
| 8 | F | 64 | 84 | III |
| 9 | M | 53 | 97 | II |
| 10 | M | 78 | 8 | IV |
| 11 | M | 77 | 68 | IV |
| 12 | F | 60 | 168 | III |
| 13 | M | 76 | 3 | IV |
| 14 | F | 73 | 200 | IV |
| 15 | M | 79 | 3 | II |

**Table III** Comparison of results of the *in vitro* assay with *in vivo* response

| Patient | In vitro *assay result (TCV; %)* Chl | 4-Cy | Vc | Pred | *Drugs given* in vivo | *Minimum TCV(%)** | *Response* in vitro†/in vivo |
|---|---|---|---|---|---|---|---|
| 1a | 31 | 10 | 103 | 70 | Cy, Pred | 10 | S/S |
| 2 | 30 | 34 | 33 | 12 | Cy, Vc, Pred | 12 | S/S |
| 3 | 13 | 21 | 15 | 24 | Cy, Vc, Pred | 15 | S/S |
| 1b | 18 | 2 | 75 | 106 | Chl, Pred | 18 | S/S |
| 4 | 26 | 28 | 43 | 51 | Cy, Vc, Pred | 28‡ | S/R |
| 5a | 28 | 53 | 39 | 50 | Chl, Vc, Pred | 28‡ | S/R |
| 6a | 80 | 31 | 55 | 81 | Cy, Vc, Pred | 31‡ | R/R |
| 5b | 39 | 61 | 33 | 71 | Cy, Vc, Pred | 33‡ | R/R |
| 7 | 100 | 31 | 31 | 78 | Chl, Vc, Pred | 31 | R/R |
| 8 | 40 | 63 | 6 | 32 | Pred | 32 | R/R |
| 9 | 83 | 90 | 62 | 32 | Cy, Vc, Pred | 32 | R/R |
| 10 | 52 | 66 | 38 | 68 | Chl, Vc, Pred | 38 | R/R |
| 11 | 46 | 68 | 40 | 42 | Chl, Pred | 42 | R/R |
| 12a | 102 | 96 | 37 | 42 | Chl, Pred | 42 | R/R |
| b | 102 | 96 | 37 | 42 | Cy, Pred | 42 | R/R |
| 13 | 29 | 57 | 8 | 63 | Pred | 63 | R/R |
| 14a | 86 | 96 | 60 | 70 | Chl, Pred | 70 | R/R |
| b | 86 | 58 | 41 | 72 | Pred | 72 | R/R |
| 15 | 74 | 87 | 66 | 73 | Pred | 73 | R/R |
| 1c | 24 | 9 | 72 | 78 | Pred | 78 | R/R |
| 6b | 80 | 31 | 55 | 81 | Chl, Pred | 80 | R/R |

*This value is the lowest *in vitro* assay result for the drugs given *in vivo*.
†*In vitro* response is based on a sensitive (S)/resistant (R) cut off of minimum TCV = 30%.
‡These patients achieved a clinical improvement in response to the treatment shown.

assay is a highly sensitive test of tumour chemosensitivity, its major role increasingly seems to be in the area of preclinical antineoplastic drug screening (Weisenthal, 1981; Salmon *et al.*, 1981). By contrast, tumour cell assays measure the proportion of all tumour cells killed. Thus, whilst stem cell assays may be capable of predicting potential tumour cure, tumour cell assays may correlate more satisfactorily with tumour response as observed by the clinician.

*Problems associated with dye exclusion assays*

We have followed the timing of Weisenthal *et al.*, (1983*a, c*) using a 4-day incubation period for the assay to allow for drug-damaged cells to become membrane permeable whilst avoiding excessive loss of control viability. During the 4 days, both cell proliferation and autolysis could occur but while these may present problems for other tumour systems, both are minimal for CLL using the present assay.

Since the loss of membrane integrity is a late event in cell death, this assay may require higher drug concentrations than the stem cell assay to achieve equivalent cell kills (Wiesenthal *et al.*, 1983*c*). This difference in sensitivity and the time required for loss of membrane integrity have been largely ignored in work attempting to correlate and compare the 2 types of assay (Roper & Drewinko, 1976, 1979; Bhuyan *et al.*, 1976). This has led to the clearly erroneous conclusion that dye exclusion assays are inappropriate to measure drug-induced cell kill (Roper & Drewinko, 1979).

One other potential problem with the present assay is that some lethally-damaged cells may take longer than 4 days to lose their membrane integrity and the extent to which this occurs may differ for each drug. However, for each drug a number of samples have shown good sensitivity, indicating that with the drug concentrations used, many of the cells have lost their membrane integrity by 4 days. In addition, no patients who have shown *in vitro* resistance have been sensitive *in vivo* indicating again that delayed death is not a major problem.

One problem common to all *in vitro* chemosensitivity assays is that of drug activity *in vitro*. Following Volm *et al.* (1979), we have used 4-hydroperoxycyclophosphamide (4-Cy) *in vitro* to predict for Cy *in vivo*. It is very active *in vitro* (requiring only 2 $\mu g ml^{-1}$) probably due, unlike the situation *in vivo*, to little of the 4-Cy being degraded to inactive metabolites.

*Comparisons with* in vivo *response*

Considerable problems arise in the attempt to compare *in vitro* and *in vivo* data when the *in vivo* results are obtained with combination chemotherapy. Because of this, different authors have used different criteria in the assessment of true and false correlations of response (Salmon *et al.*, 1978; Weisenthal *et al.*, 1983*a*, Von Hoff *et al.*, 1981). Previously, in a preliminary report of this work, we investigated the use of the TCV averaged over the drugs given *in vivo* (Bosanquet *et al.*, 1982) but here we have used the criteria of Von Hoff *et al.* (1981) requiring only one drug of a combination to be sensitive *in vitro* for comparison with the response *in vivo*. Using these criteria, it is essential that all the drugs given in combination to the patient are tested *in vitro* and we have only attempted a comparison when this condition is fulfilled. As with most other studies we have used a cut-off between sensitive and resistant of 30% TCV, which gave us 2 false positive comparisons (sensitive *in vitro*, resistant *in vivo*). This is higher than the value which would give us no false comparisons (25% TCV, see Table III) but is preferable to the higher probability of false negative comparisons being obtained with this lower cut-off.

With the inherent errors in any *in vitro* assay, a definite cut-off line between sensitivity and resistance is likely to be of less value clinically than some form of graded probability of response. Thus we would say (Table III) that any TCV below ~25% would predict for partial or complete response, whereas a value greater than ~35% would most likely show no response or progressive disease despite chemotherapy. Intermediate values would most likely predict for clinical improvement.

The administration of drug combinations to patients who respond will always mean that it is never known which of the drugs has been active *in vivo*. However, if the *in vitro* results are shown to predict responsiveness accurately, then this lack of precise knowledge need not be a major limitation.

We have shown that the assay described here is a technically feasible approach to the *in vitro* determination of chemosensitivity in CLL and that this is in concordance with clinical response. Including the work of Weisenthal and colleagues (1983*a, b*) there are now 69 *in vitro/in vivo* comparisons or correlations of which only 4 are false (all sensitive *in vitro*, resistant *in vivo*). This compares very favourably with results obtained by the stem cell assay and suggests that this dye exclusion could be of considerable value in the prediction of tumour chemosensitivity. In addition, the assay is rapid, relatively simple to perform and requires small numbers of cells. We conclude, therefore, that this assay warrants wider investigation for the prediction of tumour chemosensitivity. To this end, we are now assessing the assay and its modified form (Weisenthal *et al.*, 1983*c*) in a broader spectrum of tumour types using both phase- and cycle-specific drugs. We are also attempting to define optimum levels of VP-16-213, nitrogen mustard ($HN_2$), 1-(2-chloroethyl)-cyclohexyl-1-nitrosourea (CCNU) and 2-deoxycoformycin for use in CLL.

We would like to thank the Leukaemia Research Fund for generously supporting this work, Ms. M. Brannan for technical assistance and Ms. C. Henderson for typing the manuscript.

### References

ALBERTS, D.S. & CHEN, H.-S.G. (1980). Tabular summary of pharmacokinetic parameters relevant to *in vitro* drug assay. *Prog. Clin. Biol. Res.*, **48**, 351.

BHUYAN, B.K., LOUGHMAN, B.E., FRASER, T.J. & DAY, K.J. (1976). Comparison of different methods of determining cell viability after exposure to cytotoxic compounds. *Exp. Cell. Res.*, **97**, 275.

BOSANQUET, A.G. & GILBY, E.D. (1982). Pharmacokinetics of oral and intravenous melphalan during routine treatment of multiple myeloma. *Eur. J. Cancer Clin. Oncol.*, **18**, 355.

BOSANQUET, A.G., BIRD, M.C. & GILBY, E.D. (1982). A four-day method *in vitro* for the prediction of tumour response to cytotoxic drugs in chronic lymphocytic leukaemia and other tumours. *Biochem. Soc. Trans.*, **10**, 505.

COURTENAY, V.D. & MILLS, J. (1978). An *in vitro* colony assay for human tumours grown in immune-suppressed mice and treated *in vivo* with cytotoxic agents. *Br. J. Cancer*, **37**, 261.

DENDY, P.P. (Ed.) (1976). *Human Tumours in Short Term Culture*. London: Academic Press.

DESAI, D.V., EZDINLI, E.Z. & STUTZMAN, L. (1970). Vincristine therapy of lymphomas and chronic lymphocytic leukaemia. *Cancer*, **26**, 352.

DURKIN, W.J., GHANTA, V.K., BALCH, C.M., DAVIS, D.W. & HIRAMOTO, R.N. (1979). A methodological approach to the prediction of anti-cancer drug effect in humans. *Cancer Res.*, **39**, 402.

GROUP FOR SENSITIVITY TESTING OF TUMORS (KSST) (1981). *In vitro* short term test to determine the resistance of human tumors to chemotherapy. *Cancer*, **48**, 2127.

HAMBURGER, A.W. (1981). Use of *in vitro* tests in predictive cancer chemotherapy. *J. Natl Cancer Inst.*, **66,** 981.

HAMBURGER, A.W. & SALMON, S.E. (1977). Primary bioassay of human tumor stem cells. *Science,* **197,** 461.

HAN, T., EZDINLI, E.Z., SHIMAOKA, K. & DESAI, D.V. (1973). Chlorambucil vs. combined chlorambucil-corticosteroid therapy in chronic lymphocytic leukemia. *Cancer,* **31,** 502.

LIEPMAN, M. & VOTAW, M.L. (1978). The treatment of chronic lymphocytic leukemia with COP chemotherapy. *Cancer,* **41,** 1664.

NELSON, R.L., DYKE, R.W. & ROOT, M.A. (1980). Comparative pharmacokinetics of vindesine, vincristine and vinblastine in patients with cancer. *Cancer Treat. Rev.,* **7** (suppl.), 17.

OKEN, M.M. & KAPLAN, M.E. (1979). Combination chemotherapy with cyclophosphamide, vincristine and prednisone in the treatment of refractory lymphocytic leukemia. *Cancer Treat. Rep.,* **63,** 441.

RAI, K.R., SAWITSKY, A., CRONKITE, E.P., CHANANA, A.D., LEVY, R.N. & PASTERNACK, B.S. (1975). Clinical staging of chronic lymphocytic leukemia. *Blood,* **46,** 219.

ROPER, P.R. & DREWINKO, B. (1976). Comparison of *in vitro* methods to determine drug-induced cell lethality. *Cancer Res.,* **36,** 2182.

ROPER, P.R. & DREWINKO, B. (1979). Cell survival following treatment with anti-tumor drugs. *Cancer Res.,* **39,** 1428.

SALMON, S.E., HAMBURGER, A.W., SOEHNLEN, B., DURIE, B.G.M., ALBERTS, D.S. & MOON, T.E. (1978). Quantitation of differential sensitivity of human-tumor stem cells to anti-cancer drugs. *N. Engl. J. Med.,* **298,** 1321.

SALMON, S.E., MEYSKENS, F.L., Jr., ALBERTS, D.S., SOEHNLEN, B. & YOUNG, L. (1981). New drugs in ovarian cancer and malignant melanoma: *in vitro* phase II screening with the human tumor stem cell assay. *Cancer Treat. Rep.,* **65,** 1.

SALMON, S.E., ALBERTS, D.S., DURIE, B.G.M., and 5 others. (1980). Clinical correlations of drug sensitivity in the human tumor stem cell assay. *Rec. Res. Cancer Res.,* **74,** 300.

SAWITSKY, A., RAI, K.R., GLIDEWELL, O. & SILVER, R.T. (1977). Comparison of daily versus intermittent chlorambucil and prednisone therapy in the treatment of patients with chronic lymphocytic leukemia. *Blood,* **50,** 1049.

TANNOCK, I. (1978). Cell kinetics and chemotherapy: a critical review. *Cancer Treat. Rep.,* **62,** 1117.

VOLM, M., WAYSS, K., KAUFMANN, M. & MATTERN, J. (1979). Pretherapeutic detection of tumour resistance and the results of tumour chemotherapy. *Eur. J. Cancer.* **15,** 983.

VON HOFF, D.D. & WEISENTHAL, L. (1980). *In vitro* methods to predict for patient response to chemotherapy. *Advan. Pharmacol. Chemother.,* **17,** 133.

VON HOFF, D.D., CASPER, J., BRADLEY, E., SANDBACH, J., JONES, D. & MAKUCH, R. (1981). Association between human tumor colony-forming assay results and response of an individual patient's tumor to chemotherapy. *Am. J. Med.,* **70,** 1027.

WEISENTHAL, L.M. (1981). *In vitro* assays in preclinical antineoplastic drug screening. *Semin. Oncol.,* **8,** 362.

WEISENTHAL, L.M., MARSDEN, J.A., DILL, P.L. & MACALUSO, C.K. (1983*a*). A novel dye exclusion method for testing *in vitro* chemosensitivity of human tumors. *Cancer Res.,* **43,** 749.

WEISENTHAL, L.M., LALUDE, A.O'T. & MILLER, J.B. (1983*b*). *In vitro* chemosensitivity of human bladder cancer. *Cancer* (in press).

WEISENTHAL, L.M., DILL, P.L., KURNICK, N.B. & LIPPMAN, M.E. (1983*c*). Comparison of dye exclusion assays with a clonogenic assay in the determination of drug-induced cytotoxicity. *Cancer Res.,* **43,** 258.